# Case report: Radiofrequency ablation combined with biopsy for Cushing’s syndrome due to ectopic ACTH lesions in the lung

**DOI:** 10.3389/fonc.2022.1059308

**Published:** 2022-11-18

**Authors:** Xiao Zhang, Liangliang Meng, Yueyong Xiao, Zenan Chen

**Affiliations:** ^1^ Department of Radiology, The First Medical Center, Chinese People's Liberation Army (PLA) General Hospital, Beijing, China; ^2^ Department of Radiology, Chinese People's Armed Police (PAP) Force Hospital of Beijing, Beijing, China

**Keywords:** radiofrequency ablation, biopsy, Cushing’s syndrome, ectopic, ACTH

## Abstract

Lung carcinoid tumor is one of the major tumors causing ectopic ACTH syndrome, and the most common clinical treatment is surgical resection of the lesion. We herein report a suspected pulmonary carcinoid tumor with difficulty in surgical resection and poor response to drug therapy, which was successfully treated with radiofrequency ablation combined with intraoperative biopsy of the lesion. A 48-year-old female patient, with hypercortisolism (reddening of the face, full moon face, hirsutism, acne, and weight gain) detected three months ago. Small and high-dose dexamethasone suppression tests were not suppressed, Cushing’s syndrome was under consideration. PET-CT examination suggested mild FDG uptake in two nodules in the anterior basal segment of the lower lobe of the right lung, the possibility of ectopic ACTH lesions was considered because of the clinical presentation. Due to difficult surgical approach of the lesion, high risk of surgery and the patient’s anxiety, CT-guided thermal ablation combined with puncture biopsy was considered to treat the lesions. Image-guided thermal ablation can effectively inactivate ectopic ACTH lesions in the lung, rapidly improve the symptoms of high cortisol, and can be combined with biopsy for pathologic diagnosis. Therefore, this technique can be considered for treating pulmonary ACTH lesions that are difficult to resect surgically.

## Introduction

Adrenocorticotropic hormone(ACTH)-dependent Cushing’s syndrome accounts for approximately 80-85% of Cushing’s syndrome, of which approximately 20% is caused by ectopic ACTH syndrome (1). The majority of tumors in ectopic ACTH syndrome are neuroendocrine tumors, with the most common tumors being lung carcinoid tumors (21-39%), followed by small cell lung cancer (3-21%), thymic tumors (11%), pancreatic neuroendocrine tumors (8%), medullary thyroid carcinoma (2-11.6%), and pheochromocytoma (5.6%) (1–3). These lesions secrete adrenocorticotropic hormone (ACTH), which stimulates adrenal cortical hyperplasia and produces excess cortisol causing overt Cushing syndrome (4).

In patients who are clinically diagnosed with Cushing’s syndrome in combination with laboratory tests, hormone experiments and the evident ectopic ACTH lesions after imaging, conventional treatments such as drug therapy and surgical resection are challenging to implement (5, 6). Some techniques can be used to target the lesions and control systemic symptoms,such as radiotherapy, chemotherapy, and radionuclides (5). Still, none of the above techniques can obtain pathological tissue for pathologic diagnosis. Imaging-guided radiofrequency ablation has been applied to the treatment of tumors in various parts of the body, which has the advantages of precise localization, exact efficacy, minimal trauma, puncture biopsy can precisely cut and take the material for small lesions (7–9). Radiofrequency ablation with simultaneous biopsy for ectopic ACTH lesions in the lung has not been reported in the literature before.

## Patient description

This retrospective study was approved by the Ethics Committee of the Chinese PLA general hospital. Written informed consent was provided for this study by the patient.

The female patient, 48 years old, was found to have a reddish complexion, full moon face, increased fine hair, thin skin three months ago, weight gain of 5kg in the last month, hypertension (132/87mmHg), the presence of recurrent sleep disturbances and emotional instability in the patient.

Laboratory tests revealed hypokalemia (blood potassium 2.26-2.47 mmol/L), decreased bone metabolism (osteocalcin 9.00 ng/ml), vitamin D deficiency (Vit D 10.6 ng/ml). Then anxiety disorder was considered after the completion of relevant tests.

The patient was admitted with elevated serum cortisol (0AM:618.96 nmol/L, 8AM:637.17 nmol/L,4PM:562.20 nmol/L) and elevated ACTH levels (0AM:19.6 pmol/L, 8AM:20.7 pmol/L,4PM:19.9 pmol/L) on laboratory tests. Urine over 24 hours to measure free cortisol (2178.9 nmol), ACTH rhythms disappeared and were not suppressed by the low-dose dexamethasone suppression test (ACTH25.1 pmol/L, serum cortisol 756.42 nmol/L) or the high-dose dexamethasone suppression test (ACTH16.4 pmol/L, serum cortisol 402.35 nmol/L). The consideration of ectopic ACTH syndrome was indicated.

**Figure 1:**
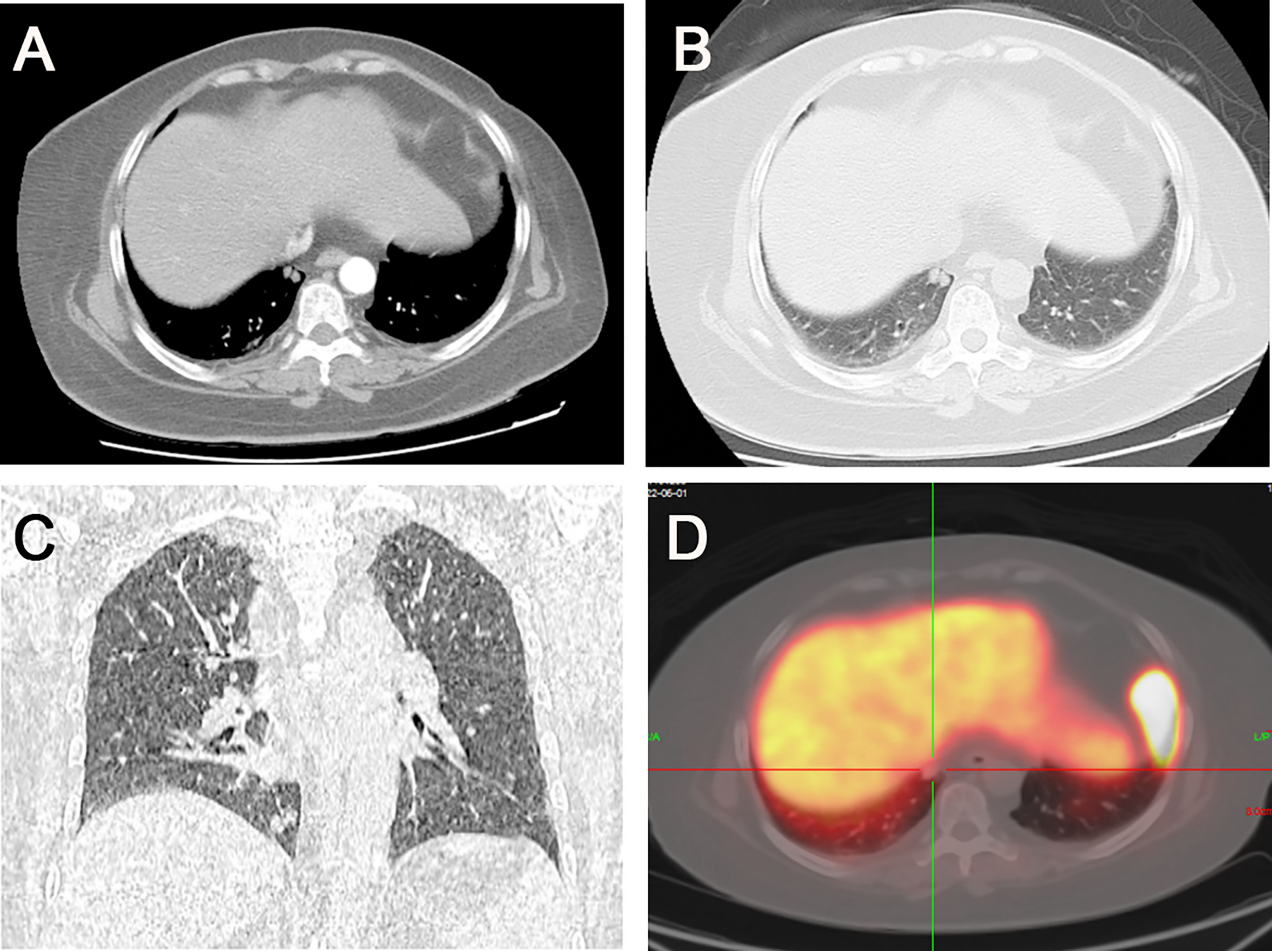
**(A)** Two rounded nodules in the right lower lobe of the lung, with well-defined borders and a diameter of about 6 mm. **(B)** Significant enhancement of the nodules in the arterial phase. **(C)** Coronal image showing the location of the nodules near the heart and diaphragm. **(D)** ^68^Ga- DOTATATE PET-CT did not show significant drug uptake.

The patient underwent an enhanced CT chest-scan, which showed a nodular shadow in the basal segment of the right lower lobe with significant enhancement. Meanwhile, chest images showed only slight thickening of the bilateral adrenal glands without obvious signs of hyperplasia. The patient underwent ^68^Ga- DOTATATE (**Figure 1**), and ^18^F-FDG PET-CT. ^68^Ga- DOTATATE PET-CT did not show significant uptake while ^18^F-FDG PET-CT showed mild radiotracer uptake (standard uptake value 2.2 g/ml).

Considering laboratory tests, typical clinical symptoms and complex location that is not suitable for surgery, after multidisciplinary discussions, the decision was made to perform radiofrequency ablation and biopsy to treat the lesion and confirm whether it was an ESA.

Symptomatic treatment, potassium supplementation, and antidepressant therapy were administered according to the patient’s clinical symptoms, but the clinical symptoms did not improve. Due to the complex location of the lesion, surgical resection was more traumatic, and the patient resisted; tracheoscopy was challenging to reach the location of the lesion for biopsy. Informed consent was obtained after consultation with the patient and family, CT-guided radiofrequency ablation combined with biopsy was used to treat the lesion. Because of the patient’s obese body size (158 cm, 86 kg), a Big bore CT (PHILIPS, Big bore 85 CM) was used as the guiding device, along with a Medtronic radiofrequency ablation system, the matching ablation probes and Bard biopsy needle kit.

**Figure 2:**
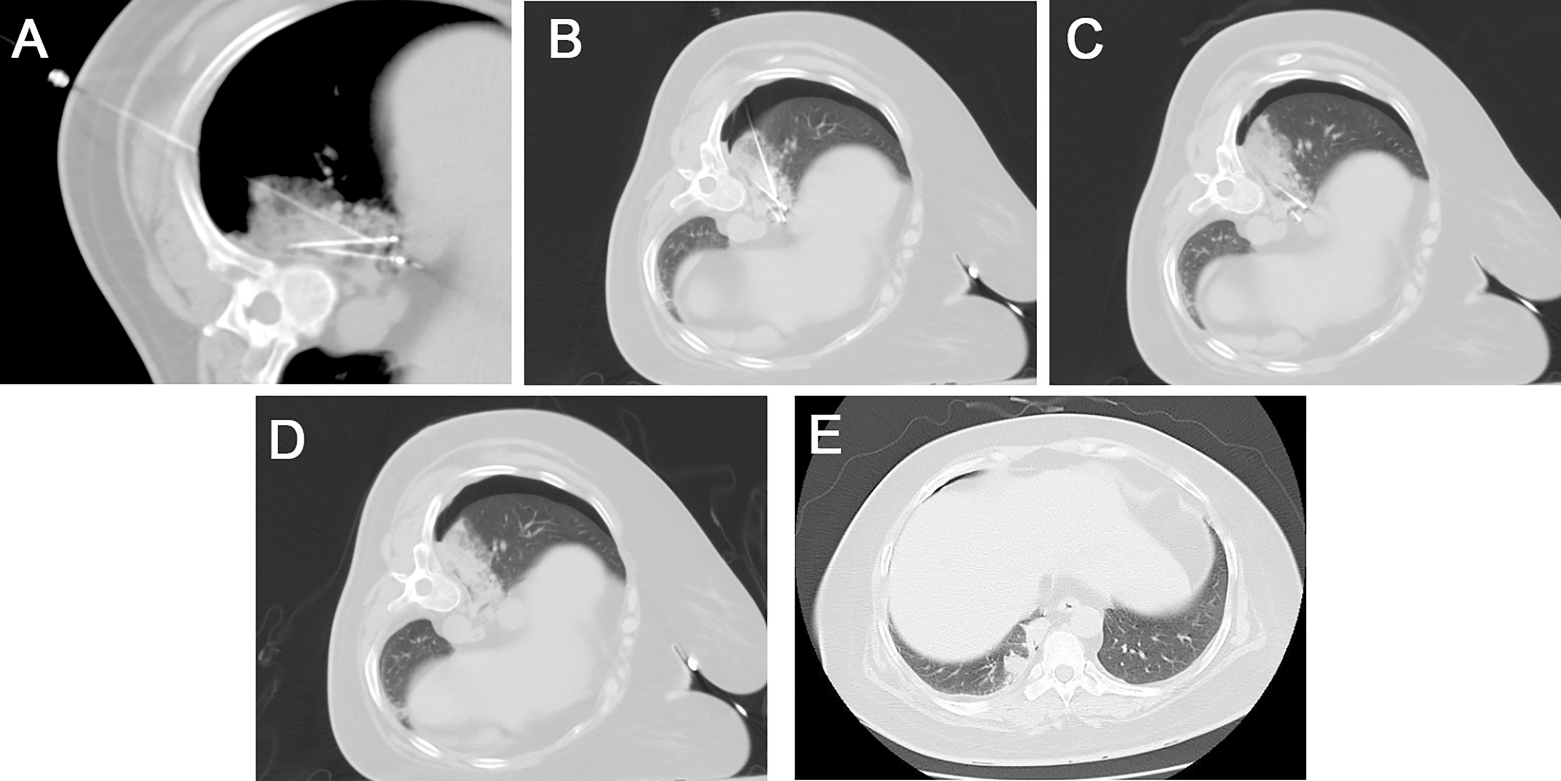
**(A)** Under CT guidance, the radiofrequency ablation probes (long arrow) were placed for the two lesions, and local nerve block anesthesia (short arrow) was performed using a 21G Chiba needle to the diaphragm. **(B)** A semi-automatic biopsy was used to obtain the tumor tissue from one of the lesions. **(C)** A scan was performed after 12 min of ablation with a power of 30 w, showing the formation of a halo sign around the lesion. **(D)** After removing the needle and probes, the CT scan showed substantial localized changes in the ablation zone. A small amount of bleeding from the puncture needle tract with a small amount of pneumothorax on the right side. **(E)** A follow-up one week after the operation showed a well-defined solid area in the ablation zone.

The patient was placed in a lateral position, then the needle was inserted in the posterior back. The ablation needle and needle sheath were guided to the lesion under CT guidance using a stepwise method. The ablation treatment was performed with a power of 30w after the position was satisfactory and the local temperature reached 80-110°C. After 3 min, the biopsy needle was implanted through the matched sheath for tissue cutting. The ablation situation was observed by scanning every 5 min during the ablation period. The needle was withdrawn after the ablation was satisfactory (the width of the surrounding halo sign around the lesion >the width after adequate ablation (width of the halo sign around the lesion >5 mm), the needle was withdrawn. The needle tract was ablated and blocked by injecting a mixture of snake venom hemagglutinin injection (Sounaswe, 1 ml) and an absorbable gelatin sponge (Geiloam, 100 mg) through the needle sheath (**Figure 2**). The patient’s vital signs, such as heart rate, blood pressure, and oxygen, were closely monitored during and after surgery. A postoperative CT scan of the chest was performed to observe complications such as bleeding, pneumothorax, and air embolism. Postoperative hemostatic drugs, analgesic and anti-inflammatory drugs were routinely used.

Postoperative blood laboratory tests performed at different time points revealed a significant decrease in cortisol and ACTH levels compared to preoperative levels (**Table 1**), and the patient’s circadian rhythm returned; CT examinations performed at 24 h and one week postoperatively showed satisfactory ablation of the lesion, solid localized lesions and surrounding lung tissue. The puncture pathology results showed: (right lower lobe of the lung) punctured lung tissue, small focal alveolar epithelial hyperplasia significant with mild atypical hyperplasia, a few histiocytic aggregates were seen around. Immunohistochemical results: Ki-67 (+3%), ALK (Ventana) (-), NapsinA (+), CK7 (+), TTF-1 (+), CD68 (histiocytes +), ACTH (-).

**Table 1:** Changes in indicators related to Cushing's syndrome in patients after surgery.

	Serum cortisol (nmol/L)			ACTH (pmol/L)			UFC (nmol/24h)
	0AM	8AM	4PM	0AM	8AM	4PM	
Preoperative	618.96	637.17	562.20	19.6	20.7	19.9	3559.5
5 hours postoperatively	113.85	140.56	85.49	6.76	7.46	7.57	216.1
5 days postoperatively	152.43	441.63	264.85	9.34	10.2	10.9	1499.3

ACTH, adreno-cortico-tropic-hormone; UFC, urinary free cortisol.

Based on the patient’s laboratory findings, the dosage of subsequent therapeutic drugs was gradually reduced, and the patient’s clinical condition gradually improved.

The presentation of the case report is in line with CARE guidelines (10).

## Results

CT-guided radiofrequency ablation and biopsy of the lesion were successfully performed. The postoperative follow-up showed good inactivation of the lesion, significant decrease in ACTH and cortisol levels, restoration of ACTH-F rhythm, and improvement of clinical symptoms. The patient has now been discharged from the hospital with recovery. The pathologic findings do not currently support the diagnosis of lung carcinoid. However, considering the clinical manifestations, laboratory tests and postoperative recovery, we still believe that this is an ESA. We also wondered if the puncture obtained less tissue in order to avoid bleeding, which may have affected the acquisition of accurate pathology.

## Discussion

Ectopic ACTH syndrome (EAS) accounts for approximately 20% of ACTH-dependent Cushing’s syndrome (CS) and 10% of all types of CS. There are two types in EAS. The first one develops slowly and consists mainly of carcinoid tumor. The tumor often has small size, low degree of malignancy and long-term course (11, 12). Therefore, it may clinically present with more typical Cushing syndrome (13). The second one develops rapidly and consists mainly of small cell lung cancer. The tumor has a high degree of malignancy, a short-term course and serious condition. It does not show symptoms of typical Cushing syndrome. The tumors causing ectopic ACTH syndrome generally have a good prognosis if they are diagnosed early and resected completely. So making the clinical diagnosis early is extremely critical for the guidance of the treatment of this disease (14).Pulmonary EAS accounts for approximately 45% of all ACTH ectopic origin sites with an onset. It is often sudden, severe, and rapidly progressive. If clinical symptoms and biochemical parameters strongly suggest the possibility of EAS, it is crucial to find the ectopic source by imaging (CT, MRI/PET) for the treatment.

Currently, the best clinical treatment for EAS is surgical resection of the primary tumor that produced the ACTH, with complete remission in approximately 83% of patients (15). However, surgical resection in patients with EAS is associated with higher risks, both in terms of the impact of hypercortisolism on the surgery and the trauma to the patient in areas that are difficult to resect, such as lesions adjacent to large blood vessels in the hilum and large airways. Of course, bilateral adrenal subtotal resection can be considered, supplementing the patient with corticosteroid with corticosteroid replacement therapy. Oral medications may also be considered to block adrenocortical hormone synthesis, such as aminoglutethimide (16), ketoconazole (17). But the above two methods will lower the quality of living.

Therefore, it requires a comprehensive multidisciplinary discussion of the patient when appropriate therapy such as radiotherapy, ablation, etc. is used clinically to treat ectopic EAS lesions (18). As mentioned in PATIENT DESCRIPTION, we should comprehensively consider the patient’s condition and choose the appropriate treatment plan. If the lung lesion is considered to be an ordinary benign lesion after discussion, a different treatment plan may be given. In addition, the possibility of performing frozen pathology before ablation was discussed preoperatively. However, due to the small and deep location of the lesion, percutaneous puncture biopsy is more difficult and has a low positive rate, which may even lead to complications such as bleeding and pneumothorax, affecting the follow-up treatment of patients. Therefore, after multidisciplinary discussion and consultation with patients, we chose to perform biopsy during the ablation process to minimize the occurrence of bleeding and other complications. In fact, there was a small amount of bleeding around the lesion during the radiofrequency ablation (RFA) needle placement, which also verified our speculation.

RFA is a procedure in which an electrode probe is precisely inserted into tumor tissues under imaging guidance (19). Electromagnetic waves are emitted through a radiofrequency transmitter to produce oscillatory friction in tumor tissue cells, resulting in coagulative necrosis of tumor tissues at a temperature of 60°C-100°C. RF ablation has been widely used in the clinical treatment of tumor lesions in many body parts, such as the lung, liver, and kidney (19–21). CT-guided RFA has the following advantages (1): Precise surgical localization. For small lesions that are difficult to localize, it is necessary to expand the resection range to ensure efficacy, which causes excessive trauma and complications to patients. In contrast, the RF electrode probe can be precisely localized under CT guidance, and the error is generally less than 2mm. (2) The diameter of the RF ablation 17G electrode probe is only 1.4mm, which can be used for multiple needle punctures during the ablation of unilateral lung lesions, which is conducive to the inactivation of tumors while maximizing the protection of lung function; (3) Real-time efficacy assessment, intermittent CT scan is used during RFA, which can judge the ablation results in real-time according to the imaging characteristics and avoid excessive ablation or incomplete ablation; (4) Compared with other parenchymal organs, RFA of parenchymal lesions can be performed in the same way. Compared with RFA of other parenchymal organs, the gas around the lung lesion can restrict the heat conduction during the ablation process, and the energy can be more easily gathered inside the lesion, which makes the ablation more efficient and effective; (5) When a biopsy is required, the biopsy needle and RF probe can be placed at the same time to avoid secondary puncture injury, and the small blood vessels damaged by the puncture cut can be closed with RF ablation heating to reduce the risk of bleeding. (6) Radiofrequency ablation as a local minimally invasive ablation treatment, combined with other therapeutic methods sometimes can improve the efficacy, such as radiotherapy, holistic therapy (depending on genetic test results, choose to use chemotherapy or molecularly targeted drug therapy) and so on.

The case report we presented helped open our minds about diagnosis and treatment about ESA.

## Conclusion

In conclusion, CT-guided radiofrequency ablation can be considered for EAS patients with challenging to resect lung lesions or high surgical risk, and a biopsy can be performed at the same time to clarify the pathology; thermal ablation can also be used to treat the lesions to reduce ACTH levels and improve the safety of surgical resection. Both ablation techniques have been proven to be effective complements to surgery. In general, image-guided radiofrequency ablation combined with biopsy is an effective and safe treatment for pulmonary EAS, especially for lung lesions that conventional techniques cannot treat. It deserves the clinical promotion.

## Data availability statement

The original contributions presented in the study are included in the article/supplementary material. Further inquiries can be directed to the corresponding author.

## Ethics statement

This retrospective study was approved by the Ethics Committee of the Chinese PLA general hospital. Written informed consent was provided for this study by the patient. Written informed consent was obtained from the individual for the publication of any potentially identifiable images or data included in this article.

## Author contributions

XZ (First Author): Conceptualization, Methodology, Investigation, Validation, Formal Analysis, Project Administration, Writing - Original Draft. LM: Data Curation, Software, Visualization, Writing - Original Draft: YX (Corresponding Author): Funding Acquisition, Resources, Supervision, Writing - Review and Editing. ZC: Validation, Writing - Review and Editing. All authors contributed to the article and approved the submitted version.

## Funding

This research was supported in part by research grant from the PLA Health Care Project of China (No. 22BJZ18).

## Conflict of interest

The authors declare that the research was conducted in the absence of any commercial or financial relationships that could be construed as a potential conflict of interest.

## Publisher’s note

All claims expressed in this article are solely those of the authors and do not necessarily represent those of their affiliated organizations, or those of the publisher, the editors and the reviewers. Any product that may be evaluated in this article, or claim that may be made by its manufacturer, is not guaranteed or endorsed by the publisher.
